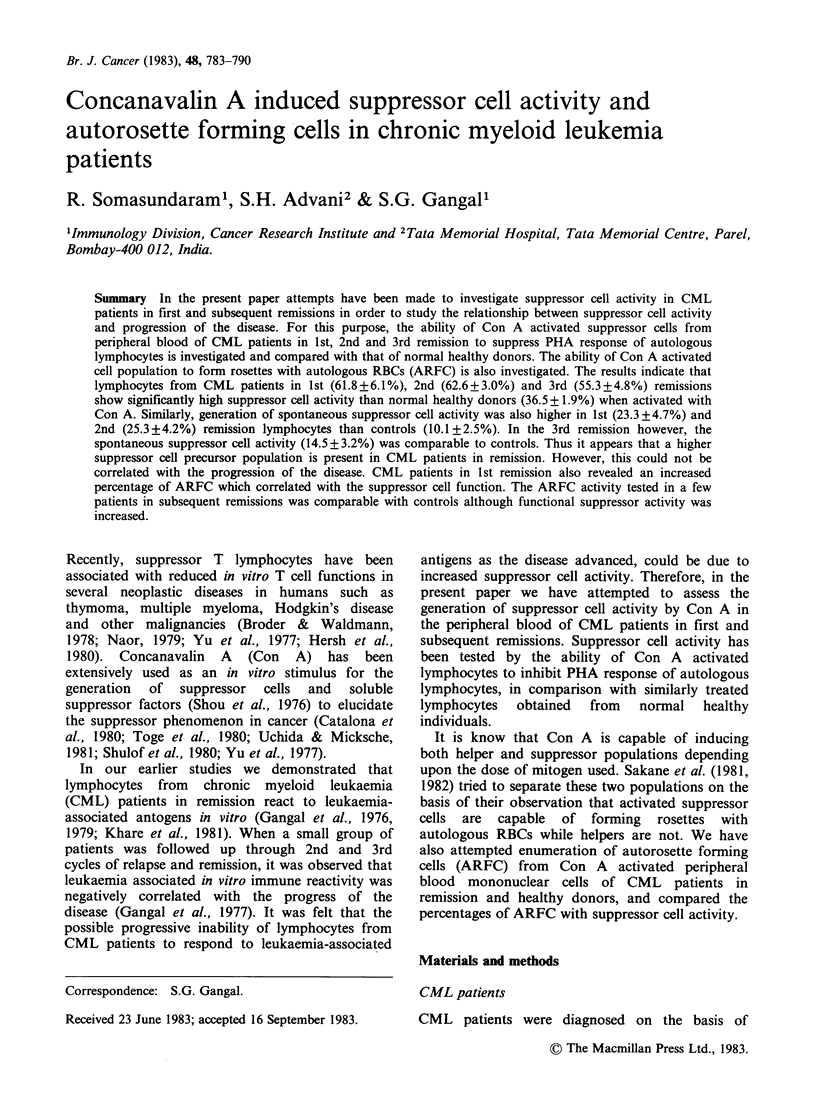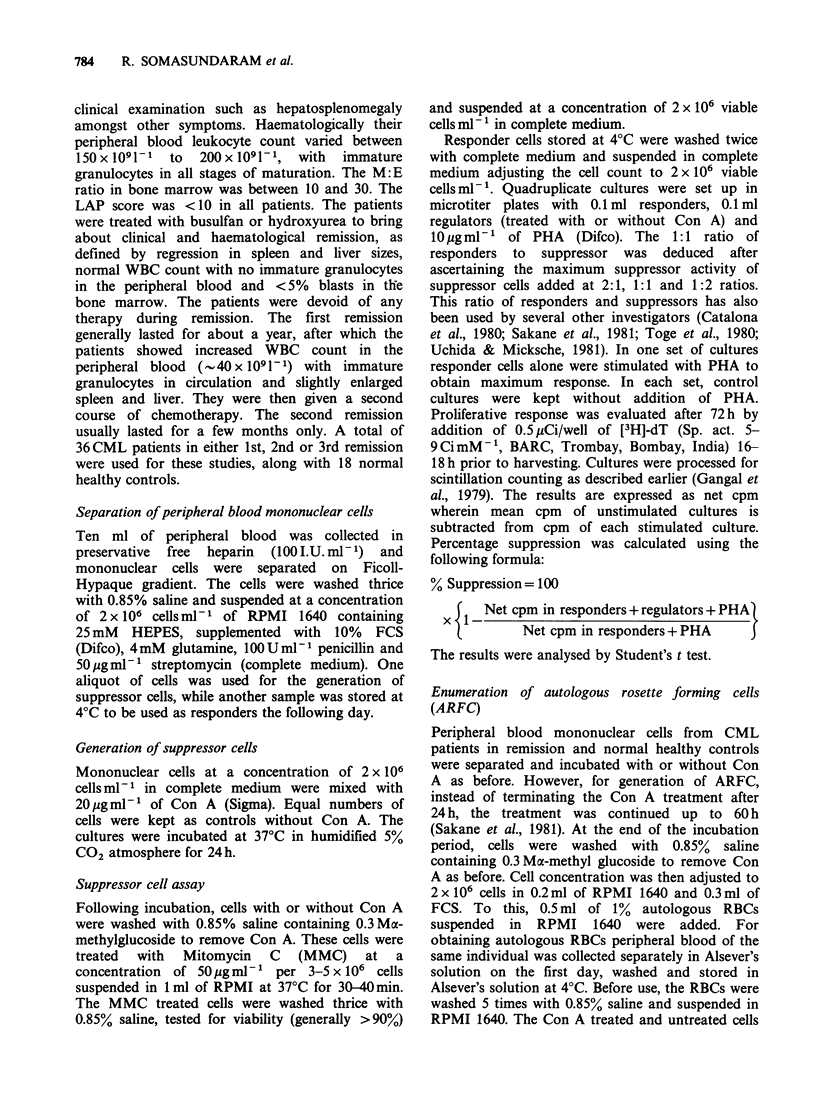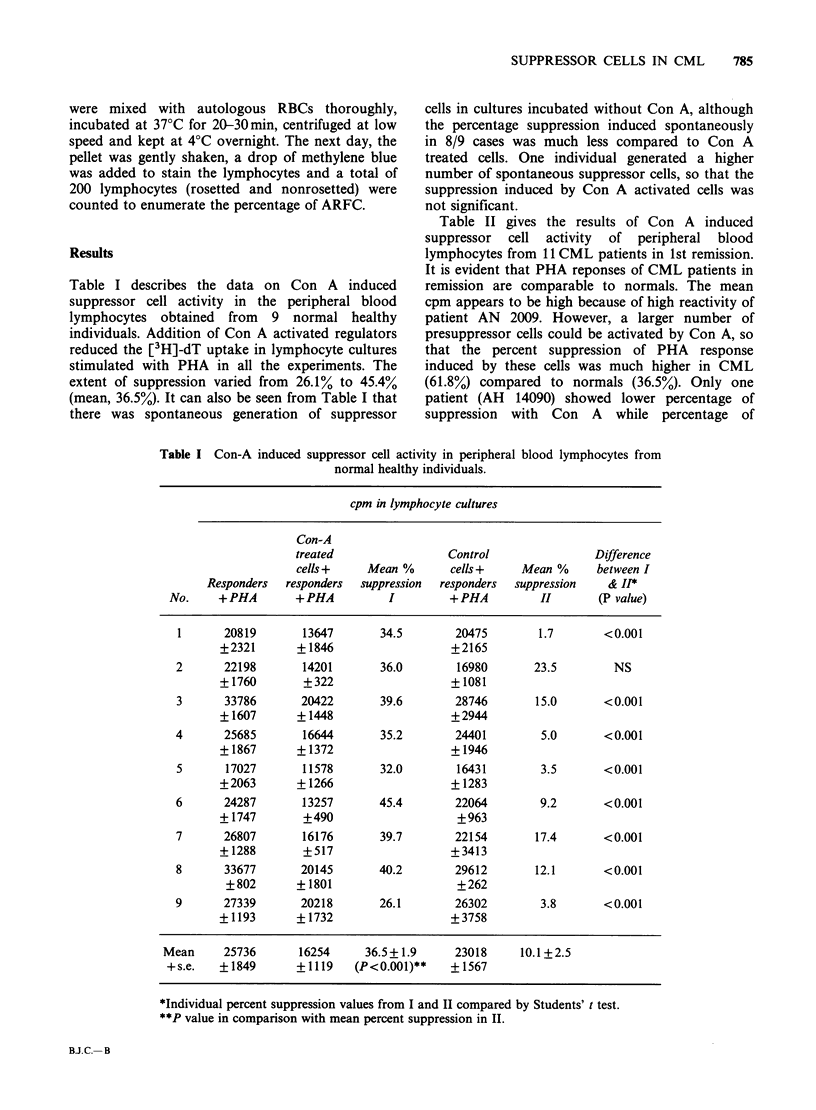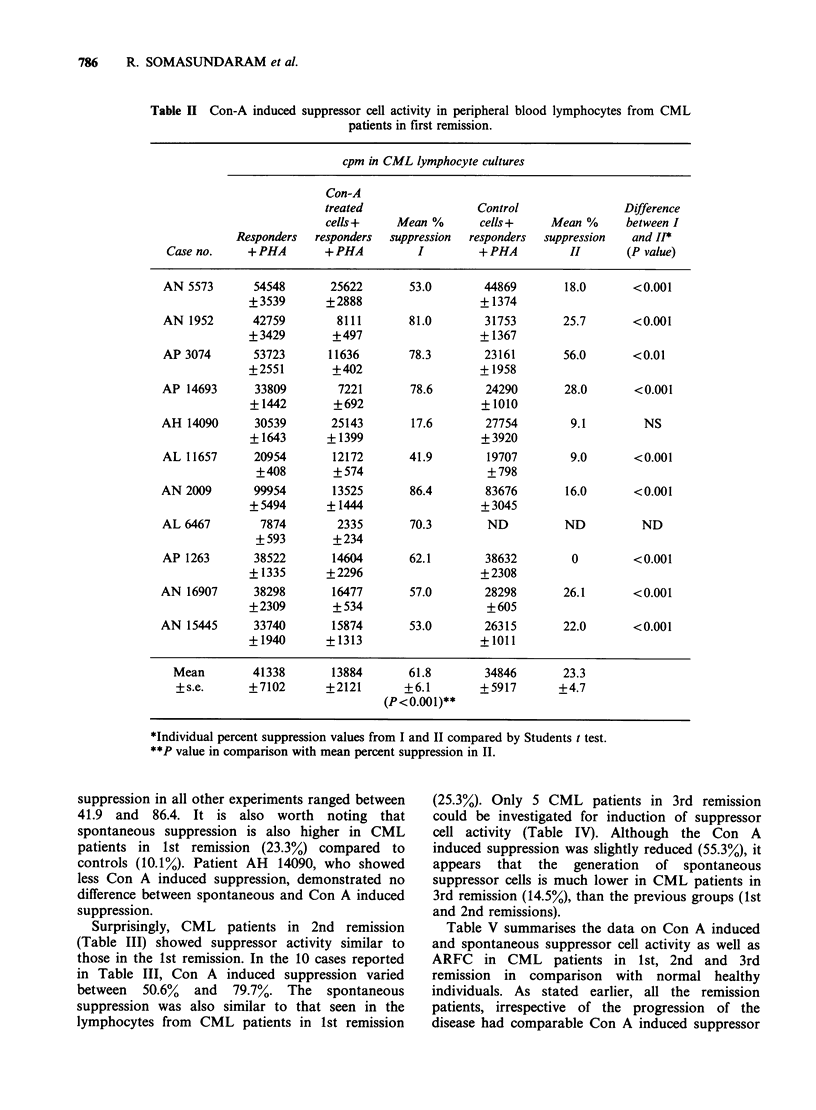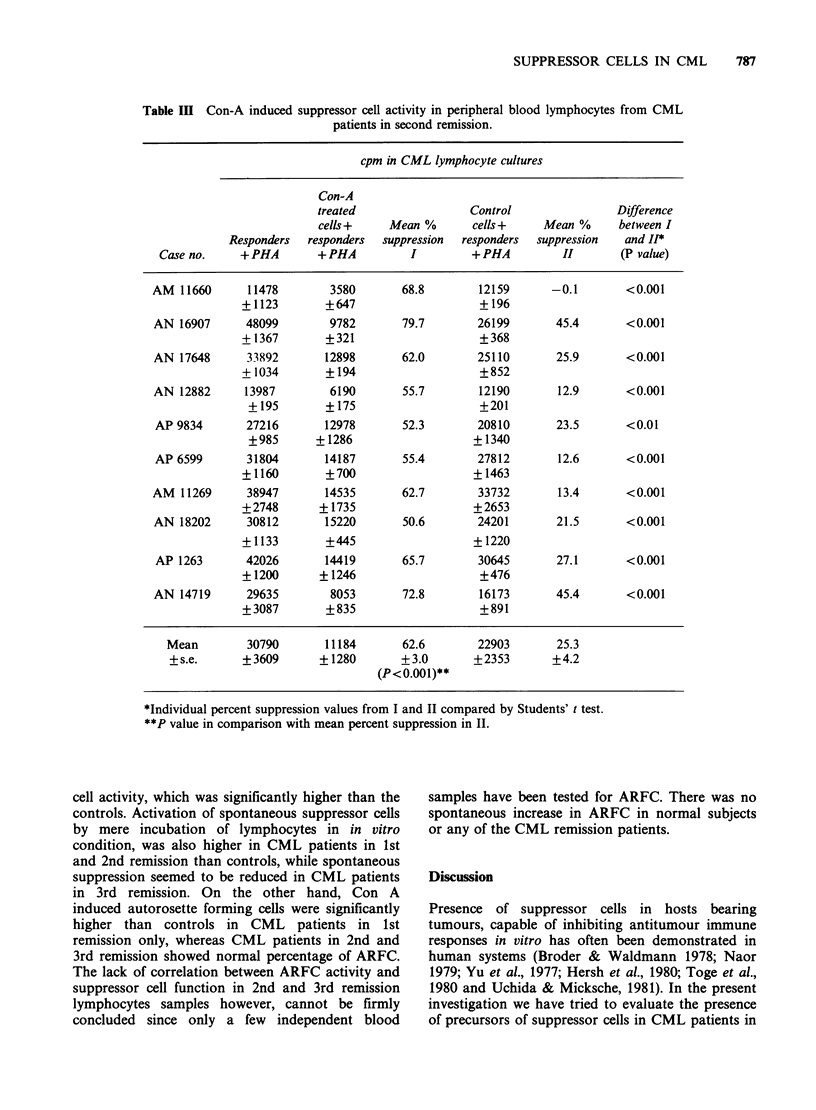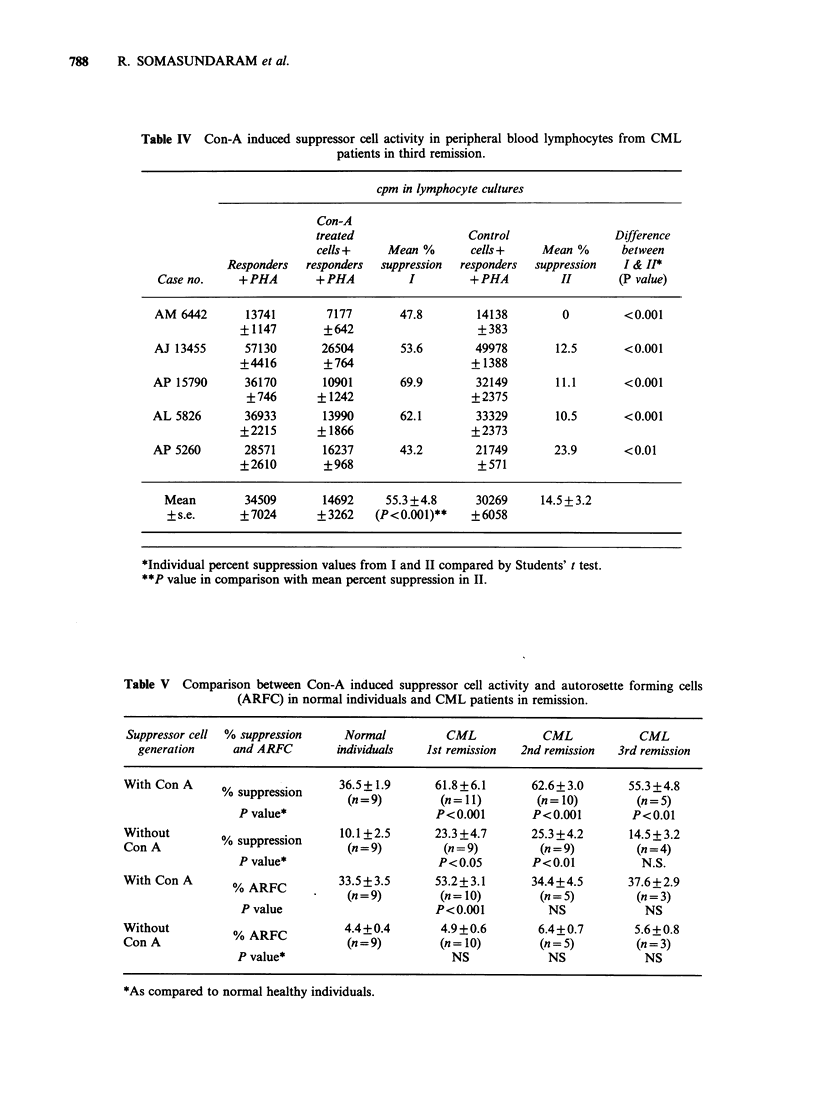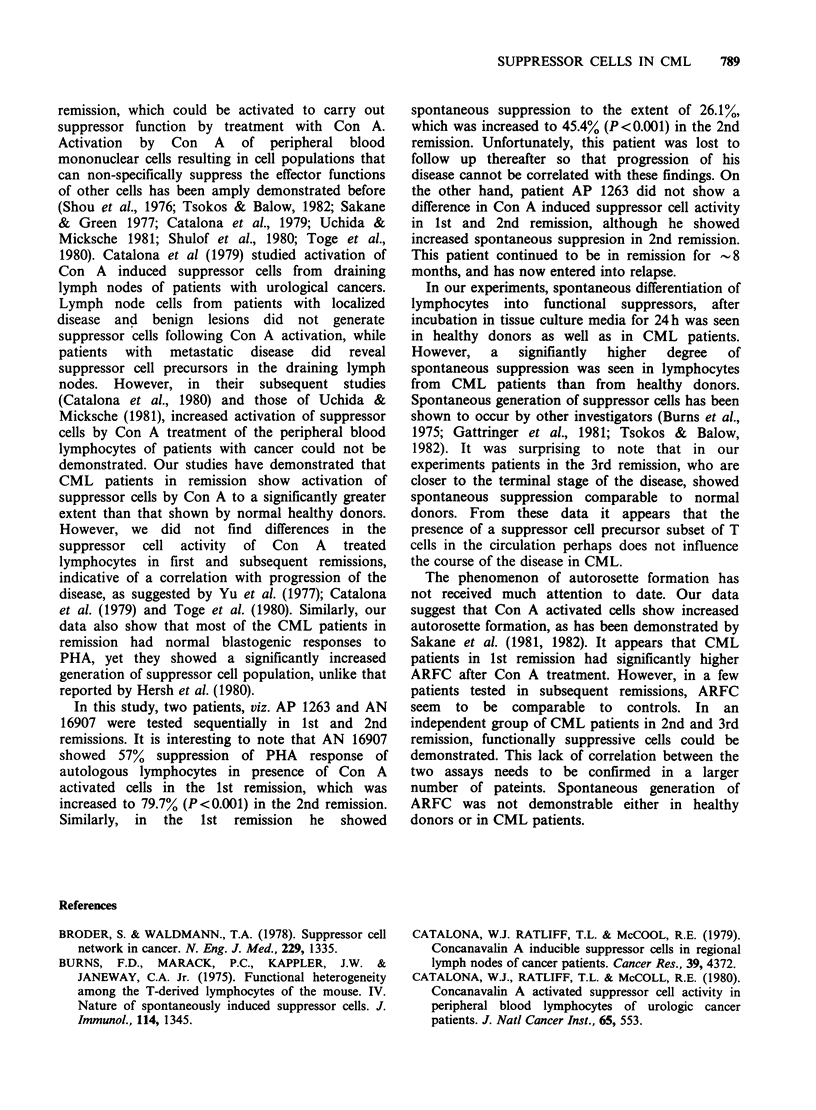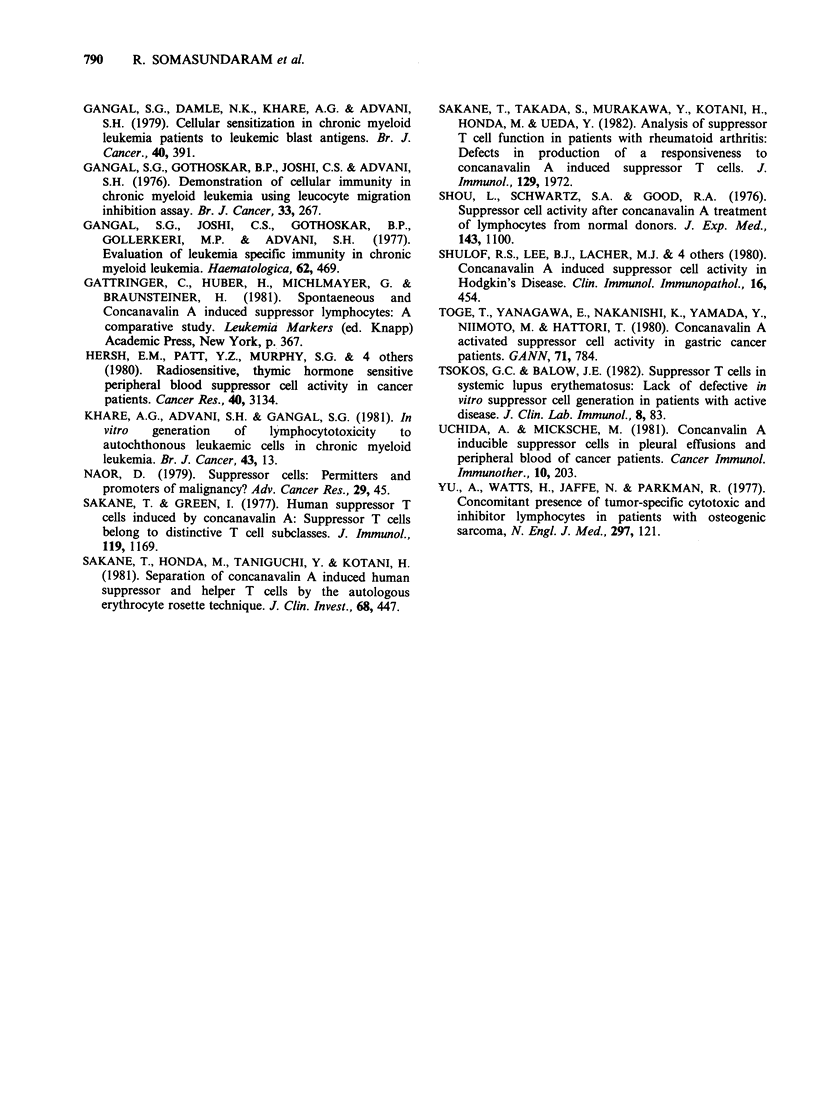# Concanavalin A induced suppressor cell activity and autorosette forming cells in chronic myeloid leukemia patients.

**DOI:** 10.1038/bjc.1983.267

**Published:** 1983-12

**Authors:** R. Somasundaram, S. H. Advani, S. G. Gangal

## Abstract

In the present paper attempts have been made to investigate suppressor cell activity in CML patients in first and subsequent remissions in order to study the relationship between suppressor cell activity and progression of the disease. For this purpose, the ability of Con A activated suppressor cells from peripheral blood of CML patients in 1st, 2nd and 3rd remission to suppress PHA response of autologous lymphocytes is investigated and compared with that of normal healthy donors. The ability of Con A activated cell population to form rosettes with autologous RBCs (ARFC) is also investigated. The results indicate that lymphocytes from CML patients in 1st (61.8 +/- 6.1%), 2nd (62.6 +/- 3.0%) and 3rd (55.3 +/- 4.8%) remissions show significantly high suppressor cell activity than normal healthy donors (36.5 +/- 1.9%) when activated with Con A. Similarly, generation of spontaneous suppressor cell activity was also higher in 1st (23.3 +/- 4.7%) and 2nd (25.3 +/- 4.2%) remission lymphocytes than controls (10.1 +/- 2.5%). In the 3rd remission however, the spontaneous suppressor cell activity (14.5 +/- 3.2%) was comparable to controls. Thus it appears that a higher suppressor cell precursor population is present in CML patients in remission. However, this could not be correlated with the progression of the disease. CML patients in 1st remission also revealed an increased percentage of ARFC which correlated with the suppressor cell function. The ARFC activity tested in a few patients in subsequent remissions was comparable with controls although functional suppressor activity was increased.


*Br. J. Cancer* (1983), **48**, 783–790

### Concanavalin A induced suppressor cell activity and autorosette forming cells in chronic myeloid leukemia patients

R. Somasundaram[1], S.H. Advani[2] & S.G. Gangal[1]

[1]*Immunology Division, Cancer Research Institute and* [2]*Tata Memorial Hospital, Tata Memorial Centre, Parel, Bombay-400 012, India.*

**Summary** In the present paper attempts have been made to investigate suppressor cell activity in CML patients in first and subsequent remissions in order to study the relationship between suppressor cell activity and progression of the disease. For this purpose, the ability of Con A activated suppressor cells from peripheral blood of CML patients in 1st, 2nd and 3rd remission to suppress PHA response of autologous lymphocytes is investigated and compared with that of normal healthy donors. The ability of Con A activated cell population to form rosettes with autologous RBCs (ARFC) is also investigated. The results indicate that lymphocytes from CML patients in 1st ($61.8 \pm 6.1\%$), 2nd ($62.6 \pm 3.0\%$) and 3rd ($55.3 \pm 4.8\%$) remissions show significantly high suppressor cell activity than normal healthy donors ($36.5 \pm 1.9\%$) when activated with Con A. Similarly, generation of spontaneous suppressor cell activity was also higher in 1st ($23.3 \pm 4.7\%$) and 2nd ($25.3 \pm 4.2\%$) remission lymphocytes than controls ($10.1 \pm 2.5\%$). In the 3rd remission however, the spontaneous suppressor cell activity ($14.5 \pm 3.2\%$) was comparable to controls. Thus it appears that a higher suppressor cell precursor population is present in CML patients in remission. However, this could not be correlated with the progression of the disease. CML patients in 1st remission also revealed an increased percentage of ARFC which correlated with the suppressor cell function. The ARFC activity tested in a few patients in subsequent remissions was comparable with controls although functional suppressor activity was increased.

Recently, suppressor T lymphocytes have been associated with reduced *in vitro* T cell functions in several neoplastic diseases in humans such as thymoma, multiple myeloma, Hodgkin's disease and other malignancies (Broder & Waldmann, 1978; Naor, 1979; Yu *et al.*, 1977; Hersh *et al.*, 1980). Concanavalin A (Con A) has been extensively used as an *in vitro* stimulus for the generation of suppressor cells and soluble suppressor factors (Shou *et al.*, 1976) to elucidate the suppressor phenomenon in cancer (Catalona *et al.*, 1980; Toge *et al.*, 1980; Uchida & Micksche, 1981; Shulof *et al.*, 1980; Yu *et al.*, 1977).

In our earlier studies we demonstrated that lymphocytes from chronic myeloid leukaemia (CML) patients in remission react to leukaemia-associated antogens *in vitro* (Gangal *et al.*, 1976, 1979; Khare *et al.*, 1981). When a small group of patients was followed up through 2nd and 3rd cycles of relapse and remission, it was observed that leukaemia associated *in vitro* immune reactivity was negatively correlated with the progress of the disease (Gangal *et al.*, 1977). It was felt that the possible progressive inability of lymphocytes from CML patients to respond to leukaemia-associated antigens as the disease advanced, could be due to increased suppressor cell activity. Therefore, in the present paper we have attempted to assess the generation of suppressor cell activity by Con A in the peripheral blood of CML patients in first and subsequent remissions. Suppressor cell activity has been tested by the ability of Con A activated lymphocytes to inhibit PHA response of autologous lymphocytes, in comparison with similarly treated lymphocytes obtained from normal healthy individuals.

It is know that Con A is capable of inducing both helper and suppressor populations depending upon the dose of mitogen used. Sakane *et al.* (1981, 1982) tried to separate these two populations on the basis of their observation that activated suppressor cells are capable of forming rosettes with autologous RBCs while helpers are not. We have also attempted enumeration of autorosette forming cells (ARFC) from Con A activated peripheral blood mononuclear cells of CML patients in remission and healthy donors, and compared the percentages of ARFC with suppressor cell activity.

Correspondence: S.G. Gangal.

Received 23 June 1983; accepted 16 September 1983.

### Materials and methods

### *CML patients*

CML patients were diagnosed on the basis of

© The Macmillan Press Ltd., 1983.

clinical examination such as hepatosplenomegaly amongst other symptoms. Haematologically their peripheral blood leukocyte count varied between $150 \times 10^9 l^{-1}$ to $200 \times 10^9 l^{-1}$, with immature granulocytes in all stages of maturation. The M:E ratio in bone marrow was between 10 and 30. The LAP score was <10 in all patients. The patients were treated with busulfan or hydroxyurea to bring about clinical and haematological remission, as defined by regression in spleen and liver sizes, normal WBC count with no immature granulocytes in the peripheral blood and <5% blasts in the bone marrow. The patients were devoid of any therapy during remission. The first remission generally lasted for about a year, after which the patients showed increased WBC count in the peripheral blood ($\sim 40 \times 10^9 l^{-1}$) with immature granulocytes in circulation and slightly enlarged spleen and liver. They were then given a second course of chemotherapy. The second remission usually lasted for a few months only. A total of 36 CML patients in either 1st, 2nd or 3rd remission were used for these studies, along with 18 normal healthy controls.

*Separation of peripheral blood mononuclear cells*

Ten ml of peripheral blood was collected in preservative free heparin (100 I.U. $ml^{-1}$) and mononuclear cells were separated on Ficoll-Hypaque gradient. The cells were washed thrice with 0.85% saline and suspended at a concentration of $2 \times 10^6$ cells $ml^{-1}$ of RPMI 1640 containing 25 mM HEPES, supplemented with 10% FCS (Difco), 4 mM glutamine, 100 U $ml^{-1}$ penicillin and 50 $\mu g$ $ml^{-1}$ streptomycin (complete medium). One aliquot of cells was used for the generation of suppressor cells, while another sample was stored at 4°C to be used as responders the following day.

*Generation of suppressor cells*

Mononuclear cells at a concentration of $2 \times 10^6$ cells $ml^{-1}$ in complete medium were mixed with 20 $\mu g$ $ml^{-1}$ of Con A (Sigma). Equal numbers of cells were kept as controls without Con A. The cultures were incubated at 37°C in humidified 5% $CO_2$ atmosphere for 24 h.

*Suppressor cell assay*

Following incubation, cells with or without Con A were washed with 0.85% saline containing 0.3 M$\alpha$-methylglucoside to remove Con A. These cells were treated with Mitomycin C (MMC) at a concentration of 50 $\mu g$ $ml^{-1}$ per $3–5 \times 10^6$ cells suspended in 1 ml of RPMI at 37°C for 30–40 min. The MMC treated cells were washed thrice with 0.85% saline, tested for viability (generally >90%) and suspended at a concentration of $2 \times 10^6$ viable cells $ml^{-1}$ in complete medium.

Responder cells stored at 4°C were washed twice with complete medium and suspended in complete medium adjusting the cell count to $2 \times 10^6$ viable cells $ml^{-1}$. Quadruplicate cultures were set up in microtiter plates with 0.1 ml responders, 0.1 ml regulators (treated with or without Con A) and 10 $\mu g$ $ml^{-1}$ of PHA (Difco). The 1:1 ratio of responders to suppressor was deduced after ascertaining the maximum suppressor activity of suppressor cells added at 2:1, 1:1 and 1:2 ratios. This ratio of responders and suppressors has also been used by several other investigators (Catalona *et al.*, 1980; Sakane *et al.*, 1981; Toge *et al.*, 1980; Uchida & Micksche, 1981). In one set of cultures responder cells alone were stimulated with PHA to obtain maximum response. In each set, control cultures were kept without addition of PHA. Proliferative response was evaluated after 72 h by addition of 0.5 $\mu$Ci/well of [$^3$H]-dT (Sp. act. 5–9 Ci $mM^{-1}$, BARC, Trombay, Bombay, India) 16–18 h prior to harvesting. Cultures were processed for scintillation counting as described earlier (Gangal *et al.*, 1979). The results are expressed as net cpm wherein mean cpm of unstimulated cultures is subtracted from cpm of each stimulated culture. Percentage suppression was calculated using the following formula:

$$\% \text{ Suppression} = 100 \times \left\{1 - \frac{\text{Net cpm in responders} + \text{regulators} + \text{PHA}}{\text{Net cpm in responders} + \text{PHA}}\right\}$$

The results were analysed by Student's $t$ test.

*Enumeration of autologous rosette forming cells (ARFC)*

Peripheral blood mononuclear cells from CML patients in remission and normal healthy controls were separated and incubated with or without Con A as before. However, for generation of ARFC, instead of terminating the Con A treatment after 24 h, the treatment was continued up to 60 h (Sakane *et al.*, 1981). At the end of the incubation period, cells were washed with 0.85% saline containing 0.3 M$\alpha$-methyl glucoside to remove Con A as before. Cell concentration was then adjusted to $2 \times 10^6$ cells in 0.2 ml of RPMI 1640 and 0.3 ml of FCS. To this, 0.5 ml of 1% autologous RBCs suspended in RPMI 1640 were added. For obtaining autologous RBCs peripheral blood of the same individual was collected separately in Alsever's solution on the first day, washed and stored in Alsever's solution at 4°C. Before use, the RBCs were washed 5 times with 0.85% saline and suspended in RPMI 1640. The Con A treated and untreated cells were mixed with autologous RBCs thoroughly, incubated at 37°C for 20–30 min, centrifuged at low speed and kept at 4°C overnight. The next day, the pellet was gently shaken, a drop of methylene blue was added to stain the lymphocytes and a total of 200 lymphocytes (rosetted and nonrosetted) were counted to enumerate the percentage of ARFC.

### Results

Table I describes the data on Con A induced suppressor cell activity in the peripheral blood lymphocytes obtained from 9 normal healthy individuals. Addition of Con A activated regulators reduced the [$^3$H]-dT uptake in lymphocyte cultures stimulated with PHA in all the experiments. The extent of suppression varied from 26.1% to 45.4% (mean, 36.5%). It can also be seen from Table I that there was spontaneous generation of suppressor cells in cultures incubated without Con A, although the percentage suppression induced spontaneously in 8/9 cases was much less compared to Con A treated cells. One individual generated a higher number of spontaneous suppressor cells, so that the suppression induced by Con A activated cells was not significant.

Table II gives the results of Con A induced suppressor cell activity of peripheral blood lymphocytes from 11 CML patients in 1st remission. It is evident that PHA reponses of CML patients in remission are comparable to normals. The mean cpm appears to be high because of high reactivity of patient AN 2009. However, a larger number of presuppressor cells could be activated by Con A, so that the percent suppression of PHA response induced by these cells was much higher in CML (61.8%) compared to normals (36.5%). Only one patient (AH 14090) showed lower percentage of suppression with Con A while percentage of

**Table I** Con-A induced suppressor cell activity in peripheral blood lymphocytes from normal healthy individuals.

| | *cpm in lymphocyte cultures* | | | | | |
|---|---|---|---|---|---|---|
| *No.* | *Responders +PHA* | *Con-A treated cells+ responders +PHA* | *Mean % suppression I* | *Control cells+ responders +PHA* | *Mean % suppression II* | *Difference between I & II** (P *value*) |
| 1 | 20819 ±2321 | 13647 ±1846 | 34.5 | 20475 ±2165 | 1.7 | <0.001 |
| 2 | 22198 ±1760 | 14201 ±322 | 36.0 | 16980 ±1081 | 23.5 | NS |
| 3 | 33786 ±1607 | 20422 ±1448 | 39.6 | 28746 ±2944 | 15.0 | <0.001 |
| 4 | 25685 ±1867 | 16644 ±1372 | 35.2 | 24401 ±1946 | 5.0 | <0.001 |
| 5 | 17027 ±2063 | 11578 ±1266 | 32.0 | 16431 ±1283 | 3.5 | <0.001 |
| 6 | 24287 ±1747 | 13257 ±490 | 45.4 | 22064 ±963 | 9.2 | <0.001 |
| 7 | 26807 ±1288 | 16176 ±517 | 39.7 | 22154 ±3413 | 17.4 | <0.001 |
| 8 | 33677 ±802 | 20145 ±1801 | 40.2 | 29612 ±262 | 12.1 | <0.001 |
| 9 | 27339 ±1193 | 20218 ±1732 | 26.1 | 26302 ±3758 | 3.8 | <0.001 |
| Mean +s.e. | 25736 ±1849 | 16254 ±1119 | 36.5±1.9 ($P$<0.001)** | 23018 ±1567 | 10.1±2.5 | |

*Individual percent suppression values from I and II compared by Students' $t$ test.
**$P$ value in comparison with mean percent suppression in II.

**Table II** Con-A induced suppressor cell activity in peripheral blood lymphocytes from CML patients in first remission.

| | cpm in CML lymphocyte cultures | | | | | |
|---|---|---|---|---|---|---|
| *Case no.* | *Responders +PHA* | *Con-A treated cells+ responders +PHA* | *Mean % suppression I* | *Control cells+ responders +PHA* | *Mean % suppression II* | *Difference between I and II** (*P value*) |
| AN 5573 | 54548 ±3539 | 25622 ±2888 | 53.0 | 44869 ±1374 | 18.0 | <0.001 |
| AN 1952 | 42759 ±3429 | 8111 ±497 | 81.0 | 31753 ±1367 | 25.7 | <0.001 |
| AP 3074 | 53723 ±2551 | 11636 ±402 | 78.3 | 23161 ±1958 | 56.0 | <0.01 |
| AP 14693 | 33809 ±1442 | 7221 ±692 | 78.6 | 24290 ±1010 | 28.0 | <0.001 |
| AH 14090 | 30539 ±1643 | 25143 ±1399 | 17.6 | 27754 ±3920 | 9.1 | NS |
| AL 11657 | 20954 ±408 | 12172 ±574 | 41.9 | 19707 ±798 | 9.0 | <0.001 |
| AN 2009 | 99954 ±5494 | 13525 ±1444 | 86.4 | 83676 ±3045 | 16.0 | <0.001 |
| AL 6467 | 7874 ±593 | 2335 ±234 | 70.3 | ND | ND | ND |
| AP 1263 | 38522 ±1335 | 14604 ±2296 | 62.1 | 38632 ±2308 | 0 | <0.001 |
| AN 16907 | 38298 ±2309 | 16477 ±534 | 57.0 | 28298 ±605 | 26.1 | <0.001 |
| AN 15445 | 33740 ±1940 | 15874 ±1313 | 53.0 | 26315 ±1011 | 22.0 | <0.001 |
| Mean ±s.e. | 41338 ±7102 | 13884 ±2121 | 61.8 ±6.1 ($P$<0.001)** | 34846 ±5917 | 23.3 ±4.7 | |

*Individual percent suppression values from I and II compared by Students $t$ test.
**$P$ value in comparison with mean percent suppression in II.

suppression in all other experiments ranged between 41.9 and 86.4. It is also worth noting that spontaneous suppression is also higher in CML patients in 1st remission (23.3%) compared to controls (10.1%). Patient AH 14090, who showed less Con A induced suppression, demonstrated no difference between spontaneous and Con A induced suppression.

Surprisingly, CML patients in 2nd remission (Table III) showed suppressor activity similar to those in the 1st remission. In the 10 cases reported in Table III, Con A induced suppression varied between 50.6% and 79.7%. The spontaneous suppression was also similar to that seen in the lymphocytes from CML patients in 1st remission (25.3%). Only 5 CML patients in 3rd remission could be investigated for induction of suppressor cell activity (Table IV). Although the Con A induced suppression was slightly reduced (55.3%), it appears that the generation of spontaneous suppressor cells is much lower in CML patients in 3rd remission (14.5%), than the previous groups (1st and 2nd remissions).

Table V summarises the data on Con A induced and spontaneous suppressor cell activity as well as ARFC in CML patients in 1st, 2nd and 3rd remission in comparison with normal healthy individuals. As stated earlier, all the remission patients, irrespective of the progression of the disease had comparable Con A induced suppressor

**Table III** Con-A induced suppressor cell activity in peripheral blood lymphocytes from CML patients in second remission.

| | *cpm in CML lymphocyte cultures* | | | | | |
|---|---|---|---|---|---|---|
| *Case no.* | *Responders +PHA* | *Con-A treated cells+ responders +PHA* | *Mean % suppression I* | *Control cells+ responders +PHA* | *Mean % suppression II* | *Difference between I and II** (P *value*) |
| AM 11660 | 11478 ±1123 | 3580 ±647 | 68.8 | 12159 ±196 | −0.1 | <0.001 |
| AN 16907 | 48099 ±1367 | 9782 ±321 | 79.7 | 26199 ±368 | 45.4 | <0.001 |
| AN 17648 | 33892 ±1034 | 12898 ±194 | 62.0 | 25110 ±852 | 25.9 | <0.001 |
| AN 12882 | 13987 ±195 | 6190 ±175 | 55.7 | 12190 ±201 | 12.9 | <0.001 |
| AP 9834 | 27216 ±985 | 12978 ±1286 | 52.3 | 20810 ±1340 | 23.5 | <0.01 |
| AP 6599 | 31804 ±1160 | 14187 ±700 | 55.4 | 27812 ±1463 | 12.6 | <0.001 |
| AM 11269 | 38947 ±2748 | 14535 ±1735 | 62.7 | 33732 ±2653 | 13.4 | <0.001 |
| AN 18202 | 30812 ±1133 | 15220 ±445 | 50.6 | 24201 ±1220 | 21.5 | <0.001 |
| AP 1263 | 42026 ±1200 | 14419 ±1246 | 65.7 | 30645 ±476 | 27.1 | <0.001 |
| AN 14719 | 29635 ±3087 | 8053 ±835 | 72.8 | 16173 ±891 | 45.4 | <0.001 |
| Mean ±s.e. | 30790 ±3609 | 11184 ±1280 | 62.6 ±3.0 (*P*<0.001)** | 22903 ±2353 | 25.3 ±4.2 | |

*Individual percent suppression values from I and II compared by Students' *t* test.
***P* value in comparison with mean percent suppression in II.

cell activity, which was significantly higher than the controls. Activation of spontaneous suppressor cells by mere incubation of lymphocytes in *in vitro* condition, was also higher in CML patients in 1st and 2nd remission than controls, while spontaneous suppression seemed to be reduced in CML patients in 3rd remission. On the other hand, Con A induced autorosette forming cells were significantly higher than controls in CML patients in 1st remission only, whereas CML patients in 2nd and 3rd remission showed normal percentage of ARFC. The lack of correlation between ARFC activity and suppressor cell function in 2nd and 3rd remission lymphocytes samples however, cannot be firmly concluded since only a few independent blood samples have been tested for ARFC. There was no spontaneous increase in ARFC in normal subjects or any of the CML remission patients.

### Discussion

Presence of suppressor cells in hosts bearing tumours, capable of inhibiting antitumour immune responses *in vitro* has often been demonstrated in human systems (Broder & Waldmann 1978; Naor 1979; Yu *et al.*, 1977; Hersh *et al.*, 1980; Toge *et al.*, 1980 and Uchida & Micksche, 1981). In the present investigation we have tried to evaluate the presence of precursors of suppressor cells in CML patients in

**Table IV** Con-A induced suppressor cell activity in peripheral blood lymphocytes from CML patients in third remission.

| | *cpm in lymphocyte cultures* | | | | | |
|---|---|---|---|---|---|---|
| *Case no.* | *Responders +PHA* | *Con-A treated cells+ responders +PHA* | *Mean % suppression I* | *Control cells+ responders +PHA* | *Mean % suppression II* | *Difference between I & II** (P *value*) |
| AM 6442 | 13741 ±1147 | 7177 ±642 | 47.8 | 14138 ±383 | 0 | <0.001 |
| AJ 13455 | 57130 ±4416 | 26504 ±764 | 53.6 | 49978 ±1388 | 12.5 | <0.001 |
| AP 15790 | 36170 ±746 | 10901 ±1242 | 69.9 | 32149 ±2375 | 11.1 | <0.001 |
| AL 5826 | 36933 ±2215 | 13990 ±1866 | 62.1 | 33329 ±2373 | 10.5 | <0.001 |
| AP 5260 | 28571 ±2610 | 16237 ±968 | 43.2 | 21749 ±571 | 23.9 | <0.01 |
| Mean ±s.e. | 34509 ±7024 | 14692 ±3262 | 55.3±4.8 ($P<0.001$)** | 30269 ±6058 | 14.5±3.2 | |

*Individual percent suppression values from I and II compared by Students' *t* test.
***P* value in comparison with mean percent suppression in II.

**Table V** Comparison between Con-A induced suppressor cell activity and autorosette forming cells (ARFC) in normal individuals and CML patients in remission.

| *Suppressor cell generation* | *% suppression and ARFC* | *Normal individuals* | *CML 1st remission* | *CML 2nd remission* | *CML 3rd remission* |
|---|---|---|---|---|---|
| With Con A | % suppression | 36.5±1.9 ($n=9$) | 61.8±6.1 ($n=11$) | 62.6±3.0 ($n=10$) | 55.3±4.8 ($n=5$) |
| | *P* value* | | $P<0.001$ | $P<0.001$ | $P<0.01$ |
| Without Con A | % suppression | 10.1±2.5 ($n=9$) | 23.3±4.7 ($n=9$) | 25.3±4.2 ($n=9$) | 14.5±3.2 ($n=4$) |
| | *P* value* | | $P<0.05$ | $P<0.01$ | N.S. |
| With Con A | % ARFC | 33.5±3.5 ($n=9$) | 53.2±3.1 ($n=10$) | 34.4±4.5 ($n=5$) | 37.6±2.9 ($n=3$) |
| | *P* value | | $P<0.001$ | NS | NS |
| Without Con A | % ARFC | 4.4±0.4 ($n=9$) | 4.9±0.6 ($n=10$) | 6.4±0.7 ($n=5$) | 5.6±0.8 ($n=3$) |
| | *P* value* | | NS | NS | NS |

*As compared to normal healthy individuals.

remission, which could be activated to carry out suppressor function by treatment with Con A. Activation by Con A of peripheral blood mononuclear cells resulting in cell populations that can non-specifically suppress the effector functions of other cells has been amply demonstrated before (Shou *et al.*, 1976; Tsokos & Balow, 1982; Sakane & Green 1977; Catalona *et al.*, 1979; Uchida & Micksche 1981; Shulof *et al.*, 1980; Toge *et al.*, 1980). Catalona *et al* (1979) studied activation of Con A induced suppressor cells from draining lymph nodes of patients with urological cancers. Lymph node cells from patients with localized disease and benign lesions did not generate suppressor cells following Con A activation, while patients with metastatic disease did reveal suppressor cell precursors in the draining lymph nodes. However, in their subsequent studies (Catalona *et al.*, 1980) and those of Uchida & Micksche (1981), increased activation of suppressor cells by Con A treatment of the peripheral blood lymphocytes of patients with cancer could not be demonstrated. Our studies have demonstrated that CML patients in remission show activation of suppressor cells by Con A to a significantly greater extent than that shown by normal healthy donors. However, we did not find differences in the suppressor cell activity of Con A treated lymphocytes in first and subsequent remissions, indicative of a correlation with progression of the disease, as suggested by Yu *et al.* (1977); Catalona *et al.* (1979) and Toge *et al.* (1980). Similarly, our data also show that most of the CML patients in remission had normal blastogenic responses to PHA, yet they showed a significantly increased generation of suppressor cell population, unlike that reported by Hersh *et al.* (1980).

In this study, two patients, *viz.* AP 1263 and AN 16907 were tested sequentially in 1st and 2nd remissions. It is interesting to note that AN 16907 showed 57% suppression of PHA response of autologous lymphocytes in presence of Con A activated cells in the 1st remission, which was increased to 79.7% ($P<0.001$) in the 2nd remission. Similarly, in the 1st remission he showed spontaneous suppression to the extent of 26.1%, which was increased to 45.4% ($P<0.001$) in the 2nd remission. Unfortunately, this patient was lost to follow up thereafter so that progression of his disease cannot be correlated with these findings. On the other hand, patient AP 1263 did not show a difference in Con A induced suppressor cell activity in 1st and 2nd remission, although he showed increased spontaneous suppresion in 2nd remission. This patient continued to be in remission for ~8 months, and has now entered into relapse.

In our experiments, spontaneous differentiation of lymphocytes into functional suppressors, after incubation in tissue culture media for 24 h was seen in healthy donors as well as in CML patients. However, a signifiantly higher degree of spontaneous suppression was seen in lymphocytes from CML patients than from healthy donors. Spontaneous generation of suppressor cells has been shown to occur by other investigators (Burns *et al.*, 1975; Gattringer *et al.*, 1981; Tsokos & Balow, 1982). It was surprising to note that in our experiments patients in the 3rd remission, who are closer to the terminal stage of the disease, showed spontaneous suppression comparable to normal donors. From these data it appears that the presence of a suppressor cell precursor subset of T cells in the circulation perhaps does not influence the course of the disease in CML.

The phenomenon of autorosette formation has not received much attention to date. Our data suggest that Con A activated cells show increased autorosette formation, as has been demonstrated by Sakane *et al.* (1981, 1982). It appears that CML patients in 1st remission had significantly higher ARFC after Con A treatment. However, in a few patients tested in subsequent remissions, ARFC seem to be comparable to controls. In an independent group of CML patients in 2nd and 3rd remission, functionally suppressive cells could be demonstrated. This lack of correlation between the two assays needs to be confirmed in a larger number of pateints. Spontaneous generation of ARFC was not demonstrable either in healthy donors or in CML patients.

### References

BRODER, S. & WALDMANN., T.A. (1978). Suppressor cell network in cancer. *N. Eng. J. Med.*, **229**, 1335.

BURNS, F.D., MARACK, P.C., KAPPLER, J.W. & JANEWAY, C.A. Jr. (1975). Functional heterogeneity among the T-derived lymphocytes of the mouse. IV. Nature of spontaneously induced suppressor cells. *J. Immunol.*, **114**, 1345.

CATALONA, W.J. RATLIFF, T.L. & McCOOL, R.E. (1979). Concanavalin A inducible suppressor cells in regional lymph nodes of cancer patients. *Cancer Res.*, **39**, 4372.

CATALONA, W.J., RATLIFF, T.L. & McCOLL, R.E. (1980). Concanavalin A activated suppressor cell activity in peripheral blood lymphocytes of urologic cancer patients. *J. Natl Cancer Inst.*, **65**, 553.

GANGAL, S.G., DAMLE, N.K., KHARE, A.G. & ADVANI, S.H. (1979). Cellular sensitization in chronic myeloid leukemia patients to leukemic blast antigens. *Br. J. Cancer.*, **40,** 391.

GANGAL, S.G., GOTHOSKAR, B.P., JOSHI, C.S. & ADVANI, S.H. (1976). Demonstration of cellular immunity in chronic myeloid leukemia using leucocyte migration inhibition assay. *Br. J. Cancer,* **33,** 267.

GANGAL, S.G., JOSHI, C.S., GOTHOSKAR, B.P., GOLLERKERI, M.P. & ADVANI, S.H. (1977). Evaluation of leukemia specific immunity in chronic myeloid leukemia. *Haematologica,* **62,** 469.

GATTRINGER, C., HUBER, H., MICHLMAYER, G. & BRAUNSTEINER, H. (1981). Spontaeneous and Concanavalin A induced suppressor lymphocytes: A comparative study. *Leukemia Markers* (ed. Knapp) Academic Press, New York, p. 367.

HERSH, E.M., PATT, Y.Z., MURPHY, S.G. & 4 others (1980). Radiosensitive, thymic hormone sensitive peripheral blood suppressor cell activity in cancer patients. *Cancer Res.,* **40,** 3134.

KHARE, A.G., ADVANI, S.H. & GANGAL, S.G. (1981). *In vitro* generation of lymphocytotoxicity to autochthonous leukaemic cells in chronic myeloid leukemia. *Br. J. Cancer,* **43,** 13.

NAOR, D. (1979). Suppressor cells: Permitters and promoters of malignancy? *Adv. Cancer Res.,* **29,** 45.

SAKANE, T. & GREEN, I. (1977). Human suppressor T cells induced by concanavalin A: Suppressor T cells belong to distinctive T cell subclasses. *J. Immunol.,* **119,** 1169.

SAKANE, T., HONDA, M., TANIGUCHI, Y. & KOTANI, H. (1981). Separation of concanavalin A induced human suppressor and helper T cells by the autologous erythrocyte rosette technique. *J. Clin. Invest.,* **68,** 447.

SAKANE, T., TAKADA, S., MURAKAWA, Y., KOTANI, H., HONDA, M. & UEDA, Y. (1982). Analysis of suppressor T cell function in patients with rheumatoid arthritis: Defects in production of a responsiveness to concanavalin A induced suppressor T cells. *J. Immunol.,* **129,** 1972.

SHOU, L., SCHWARTZ, S.A. & GOOD, R.A. (1976). Suppressor cell activity after concanavalin A treatment of lymphocytes from normal donors. *J. Exp. Med.,* **143,** 1100.

SHULOF, R.S., LEE, B.J., LACHER, M.J. & 4 others (1980). Concanavalin A induced suppressor cell activity in Hodgkin's Disease. *Clin. Immunol. Immunopathol.,* **16,** 454.

TOGE, T., YANAGAWA, E., NAKANISHI, K., YAMADA, Y., NIIMOTO, M. & HATTORI, T. (1980). Concanavalin A activated suppressor cell activity in gastric cancer patients. *GANN,* **71,** 784.

TSOKOS, G.C. & BALOW, J.E. (1982). Suppressor T cells in systemic lupus erythematosus: Lack of defective *in vitro* suppressor cell generation in patients with active disease. *J. Clin. Lab. Immunol.,* **8,** 83.

UCHIDA, A. & MICKSCHE, M. (1981). Concanvalin A inducible suppressor cells in pleural effusions and peripheral blood of cancer patients. *Cancer Immunol. Immunother.,* **10,** 203.

YU., A., WATTS, H., JAFFE, N. & PARKMAN, R. (1977). Concomitant presence of tumor-specific cytotoxic and inhibitor lymphocytes in patients with osteogenic sarcoma, *N. Engl. J. Med.,* **297,** 121.